# Comparative Evaluation of Periodontal Pathogen Load and Health in Patients Treated With Fixed Orthodontic Appliances Versus Clear Aligners: A Prospective Clinical Study

**DOI:** 10.7759/cureus.93016

**Published:** 2025-09-23

**Authors:** Sumedha Sen, Mohsin A Wani, Jasleen Kour, Shiraz Siddiqui, Shreya Chatterjee, Arti Devi, Seema Gupta

**Affiliations:** 1 Department of Orthodontics, Mithila Minority Dental College and Hospital, Darbhanga, IND; 2 Department of Orthodontics, Desh Bhagat Dental College and Hospital, Mandi Gobindgarh, IND; 3 Department of Dentistry, Shri Mata Vaishno Devi Institute of Medical Excellence, Katra, IND; 4 Department of Orthodontics, Mustafa Dental Clinic, Aligarh, IND; 5 Department of Oral Pathology and Microbiology, Institute of Technology and Science Dental College, Ghaziabad, IND; 6 Department of Orthodontics, Maharishi Markandeshwar College of Dental Sciences and Research, Ambala, IND; 7 Department of Orthodontics, Kothiwal Dental College and Research Centre, Moradabad, IND

**Keywords:** aggregatibacter actinomycetemcomitans, fixed, orthodontic appliances, periodontal diseases, porphyromonas gingivalis

## Abstract

Introduction: Orthodontic appliances can alter the oral microbial ecosystem and predispose patients to plaque accumulation and periodontal inflammation. Fixed appliances introduce bracket-wire complexes that favor biofilm retention, whereas clear aligners are removable and may allow better maintenance of oral hygiene. This study aimed to compare short-term changes in clinical periodontal parameters and subgingival colonization by *Porphyromonas gingivalis* and *Aggregatibacter actinomycetemcomitans* in patients undergoing orthodontic treatment with fixed appliances or clear aligners.

Materials and methods: This prospective study was conducted in the Department of Orthodontics, where 40 healthy adults aged 18-30 years were enrolled and equally divided into two groups: fixed appliances (n=20) and clear aligners (n=20). Baseline professional prophylaxis was administered to all patients before appliance placement. Clinical assessments, including plaque index, bleeding on probing, and probing depth, were recorded at baseline (T0) and after three months (T1). Subgingival plaque samples were collected from the second premolars and lateral incisors, and bacterial loads of *P. gingivalis* and *A. actinomycetemcomitans* were quantified by quantitative polymerase chain reaction (qPCR). Statistical analyses included independent t-tests for clinical parameters and Mann-Whitney U tests for bacterial loads, with significance set at p<0.05.

Results: At baseline, the two groups were comparable with no significant differences in clinical parameters or microbial counts. After three months, the fixed appliance group showed significantly greater deterioration in periodontal parameters than the clear aligner group. The mean increase in plaque index was 0.54±0.26 for fixed appliances versus 0.20±0.16 for clear aligners (p<0.001). Bleeding on probing and probing depth also worsened in the fixed appliance group. Microbiological analysis revealed higher bacterial loads for both pathogens in the fixed appliance group. Median *P. gingivalis* levels were 150.5×10⁴ copies/mL in fixed appliances versus 98×10⁴ copies/mL in clear aligners, while *A. Actinomycetemcomitans* was more abundant in fixed appliances, particularly at premolar sites.

Conclusion: Fixed orthodontic appliances were associated with significantly greater increases in periodontopathogen loads and deterioration in periodontal health during the early treatment period than clear aligners. Therefore, clear aligners may be a more favorable option for patients in whom periodontal preservation is a priority.

## Introduction

Orthodontic treatment has revolutionized dental care by addressing malocclusion, improving esthetics, and enhancing oral functions. With a growing demand for improved esthetics among adolescents and adults, 78.82% of Indians expressed a desire to have a pleasing smile [1]. Traditional fixed braces consisting of brackets, wires, and bands have long been the standard for correcting complex dental alignments [2]. However, the advent of clear aligners, such as Invisalign, introduced in the late 1990s, has provided a removable, aesthetically discreet alternative that appeals to patients seeking minimal visibility and comfort [3]. These thermoplastic trays are custom-fabricated using computer-aided design and manufacturing (CAD/CAM) technology, allowing sequential tooth movement without the encumbrance of fixed hardware [3,4].

Despite their benefits, orthodontic appliances can disrupt oral homeostasis, particularly affecting periodontal health. The periodontium, comprising the gums, periodontal ligament, and alveolar bone, is vulnerable to inflammation and disease owing to plaque accumulation and microbial shifts [5]. The oral cavity harbors a diverse microbiome of over 700 bacterial species that maintain a symbiotic balance under normal conditions. Dysbiosis and alterations in microbial composition can lead to gingivitis, periodontitis, caries, and enamel demineralization, exacerbated by orthodontic interventions that create niches for biofilm formation [5,6]. Fixed braces, with their irregular surfaces, promote food trapping and hinder effective brushing, fostering anaerobic environments conducive to pathogenic bacteria such as *Porphyromonas gingivalis* and *Aggregatibacter actinomycetemcomitans* [7]. In contrast, clear aligner removability facilitates routine oral hygiene, potentially mitigating these risks [8].

Numerous studies have investigated the impact of orthodontic appliances on periodontal microbiota, revealing distinct patterns between fixed braces and clear aligners [9,10]. Systematic reviews indicate that fixed appliances induce significant dysbiosis, with an increased relative abundance of gram-negative anaerobes and caries-associated species such as *Streptococcus mutans* and *Lactobacillus *[8,11]. This shift correlates with an elevated plaque index (PI), gingival index (GI), bleeding on probing (BOP), and probing depth (PD), thereby increasing the risk of periodontal inflammation and white spot lesions [5-7].

Conversely, clear aligners are associated with milder microbial perturbations [8]. Research has shown that they maintain microbial diversity and lower total bacterial loads, with stable or reduced levels of pathogens compared to the baseline. Their design allows for easy removal during meals and brushing, resulting in better periodontal indices and fewer detrimental changes [8,11]. A systematic review of eight studies involving over 500 participants confirmed that aligners reduce PI and stabilize PD and BOP, promoting a healthier microbiome that is less prone to dysbiosis [8]. However, clear aligners can accumulate biofilms on surfaces with microcracks, necessitating combined mechanical and chemical cleaning protocols [12].

Despite these insights, gaps persist in long-term data and standardized methodologies across studies, which often feature small sample sizes and short study durations. Variations in patient age, hygiene practices, and microbial sampling techniques (e.g., saliva swabs vs. subgingival plaque) complicate direct comparisons. Moreover, while clear aligners appear superior for periodontal health, their efficacy in complex cases and their overall microbial resilience require further scrutiny.

This comparative analysis aimed to quantify and compare the relative abundances of *P. gingivalis* and *A. actinomycetemcomitans* in subgingival plaque samples collected from patients undergoing orthodontic treatment with either a fixed appliance or clear aligners after a three-month treatment period. The null hypothesis for this study stated that there would be no statistically significant difference between the groups.

## Materials and methods

This prospective study was conducted in the Department of Orthodontics, Mithila Minority Dental College and Hospital, Darbhanga, India, from June 2023 to June 2024. Ethical approval was obtained from the Institutional Ethical Committee (Approval No. EC/NEW/INST/2022/4152/TH21), and the study adhered to the Declaration of Helsinki. All patients provided written informed consent prior to enrolment, after being fully informed about the study's purpose, procedures, potential risks, benefits, and their right to withdraw at any time without prejudice.

Eligible patients were adults aged 18-30 years, systemically and periodontally healthy, as confirmed by medical history and periodontal examination, including a PD of ≤3 mm, <10% sites of BOP, and no clinical attachment loss. Only patients treated with clear aligners or fixed appliances using a non-extraction approach were included. The treatment addressed minor crowding (<2 mm), deep bite, or spacing of an underlying dentoskeletal Class I pattern. Exclusion criteria included patients with systemic diseases (such as diabetes and autoimmune diseases), bone disorders, smokers, patients taking antibiotics or corticosteroids in the past three months, periodontal disease, patients who had undergone periodontal therapy in the past six months, previous orthodontic treatment, missing teeth, craniofacial anomalies, pregnant and lactating females, and those with severe malocclusions requiring extraction or surgical intervention.

The sample size was determined using G*Power software version 3.1.9.2 (Heinrich Heine University, Düsseldorf, Germany), based on the primary outcome of the plaque index. Assuming an expected mean difference of 1.35 between the clear aligner and fixed appliance groups (as referenced in prior studies), a minimum of 20 patients per group was calculated to achieve 80% statistical power with a 5% alpha error rate [13].

Oral hygiene practices were standardized via education sessions at baseline, and compliance was monitored through self-reported logs and clinical assessments to minimize variability. One week prior to treatment initiation, all patients underwent professional oral prophylaxis, including scaling, polishing, and fluoride application, to establish a uniform baseline microbial environment and reduce pre-existing plaque.

Orthodontic treatment commenced one week after prophylaxis. For Group A (fixed appliance), (n=20) brackets with McLaughlin, Bennett, Trevisi (MBT) prescription (0.022-inch slot, 3M Unitek Corp., California, USA) were bonded to the teeth using Transbond XT adhesive (also from 3M Unitek). The initial alignment was achieved using 0.014-inch nickel-titanium archwires. For Group B (clear aligners) (n=20), custom-fabricated K Aligners (K Line Europe GmbH, Düsseldorf, Germany) were used, with patients instructed to wear each aligner for 22 h per day and change trays every two weeks as per the treatment plan. The orthodontic treatment for both groups was done by an experienced orthodontist with more than five years of working experience (Sumedha Sen). 

Subgingival plaque samples were collected at baseline (one week post-prophylaxis) and after three months of treatment. Samples were obtained from the mesial and distal sites of the upper and lower second premolars and lateral incisors (16 sites per patient) using sterile Gracey curettes (Hu-Friedy, Chicago, Illinois, USA). Care was taken to isolate the sites using cotton rolls and gentle air-drying to avoid contamination. The collected plaque was immediately suspended in 1 mL of reduced transport fluid and stored at -80°C until analysis. Microbial quantification was performed using quantitative polymerase chain reaction (qPCR) with species-specific primers for *P. gingivalis *and *A. actinomycetemcomitans*, along with 16S rRNA gene sequencing for broader microbiota profiling. DNA extraction was performed using a DNA Mini Kit ( Qiagen, Hilden, Germany), and sequencing was conducted on an Illumina MiSeq platform (Illumina, headquartered in San Diego, California, USA). The bacterial loads were reported as gene copies per milliliter (copies/mL).

Clinical periodontal parameters, including PI, BOP, and PD, were assessed at baseline and three months after starting for all teeth until the second molars using a UNC-15 periodontal probe (Hu-Friedy, Chicago, Illinois, USA). PI scored plaques from 0 (none) to 3 (abundant), with site scores averaged per patient [14]. PD was used to measure sulcus depth in millimeters (mm), and the mean values were computed. After measuring the PD, the corresponding sites were inspected for the presence or absence of BOP and noted in an evaluation chart by gentle probing along the wall of the soft tissue of the gingival sulcus. To ensure consistency, assessments were conducted by two calibrated examiners under standardized conditions. To further manage confounders, dietary habits and oral hygiene were monitored via weekly diaries.

The primary outcome was the change in the relative abundance of *P. gingivalis* and *A. actinomycetemcomitans* from baseline to three months. The secondary outcomes included changes in the clinical parameters (PI, BOP, and PD). All the measurements were performed by two calibrated examiners (Jasleen Kour and Shiraz Siddiqui). Calibration involved duplicate assessments in 10 non-study patients, achieving inter-examiner reliability with intraclass correlation coefficients (ICC) >0.90 for PI and PD, and Cohen's kappa >0.85 for BOP. Microbial analyses were conducted in triplicate for qPCR to ensure reproducibility, with coefficients of variation of <5% (Shreya Chhaterjee). Data analysts (Arti Devi and Mohsin A. Wani) were blinded to group allocation to reduce bias.

Statistical analysis

Data were analyzed using Statistical Package for Social Sciences (SPSS) software (version 20, IBM Corp., Armonk, NY, USA). The normality of the data distribution for periodontal parameters (PI, BOP, and PD) and bacterial load was assessed using the Shapiro-Wilk test. As the periodontal parameters followed a normal distribution, parametric tests were employed for the analyses, whereas non-parametric methods were used for bacterial load due to their non-normal distribution. For between-group comparisons, an independent t-test was applied to evaluate the significance of the mean difference. The Mann-Whitney U test was used to analyze differences in the bacterial load between the groups. The null hypothesis assumed no significant mean difference between groups, and the significance threshold was set at p<0.05.

## Results

The study groups showed comparable demographic characteristics, with no significant differences in sex distribution (p=0.138) or mean age (23.56±4.65 vs. 21.54±5.72 years, p=0.478), as shown in Table 1.

**Table 1:** Demographic details of study population Age is presented as mean±SD, and sex distribution is presented as frequency (n) and percentage (%), where n denotes the number of patients in each group. P>0.05 indicates no statistical significance using an independent t-test for age and a chi-square test for sex distribution. SD: standard deviation

Variables	Group 1 (fixed appliance)	Group 2 (clear aligners)	Test statistics	P-value
Males, n (%)	7 (35)	9 (45)	1.56	0.138
Females, n (%)	13 (65)	11 (55)		
Age (years) mean±SD	23.56±4.65	21.54±5.72	0.38	0.478

At baseline (T0), no statistically significant differences were observed in periodontal parameters between the fixed appliance and clear aligner groups. The PI showed comparable medians (p=0.644), whereas the BOP exhibited similar means (p=0.698). PD, although it was marginally higher in the fixed appliance group, the difference was not statistically significant (p=0.143). These findings confirmed a balanced baseline periodontal status between the groups, thus validating the comparability of the study (Table 2).

**Table 2:** Comparison of periodontal parameters between study groups at baseline (T0) with an independent t-test Data are presented as mean±SD, as well as median, minimum, and maximum values. P>0.05 indicates no statistical significance using an independent t-test. PI: plaque index; BOP: bleeding on probing; PD: pocket depth; SD: standard deviation

Variables	Groups	Baseline (T0)				T statistics	P-value
		Median	Minimum	Maximum	Mean±SD		
PI (scores)	Fixed appliance	0.12	0	0.15	0.11±0.04	0.47	0.644
	Clear aligners	0.12	0	0.16	0.11±0.05		
BOP (%)	Fixed appliance	3.00	0	6.00	2.87±2.03	0.39	0.698
	Clear aligners	2.00	0	5.00	2.60±1.68		
PD (mm)	Fixed appliance	0.98	0	1.34	0.82±0.47	1.51	0.143
	Clear aligners	0.68	0	1.02	0.58±0.40		

The null hypothesis of no intergroup differences was rejected for all the parameters. The fixed appliance group demonstrated significantly greater worsening of periodontal parameters than the clear aligner group at the three-month follow-up (T1-T0). PI increased significantly in fixed appliances (mean change: 0.54±0.26 in fixed appliances vs. 0.20±0.16 in clear aligners), as did BOP and PD (1.25±0.34 mm in fixed appliances vs. 0.89±0.30 mm in clear aligners). These statistically significant differences suggest that fixed appliances exacerbate periodontal inflammation more than clear aligners (Table 3).

**Table 3:** Comparison of change in periodontal parameters from baseline (T0) to three months (T1) between study groups using an independent t-test ^*^P=0.001 indicates statistical significance using an independent t-test. Data are presented as mean±SD, as well as median, minimum, and maximum values. Mean difference calculated as mean (fixed appliance)-mean (clear aligner). PI: plaque index; BOP: bleeding on probing; PD: pocket depth; SD: standard deviation

Parameter	Groups	Median	Minimum	Maximum	Mean±SD	Mean difference	T statistics	P-value
PI (scores)	Fixed appliance	0.50	0.13	0.99	0.54±0.26	0.34	4.38	0.001^*^
	Clear aligners	0.17	-0.04	0.55	0.20±0.16			
BOP (%)	Fixed appliance	17.70	8.30	26.70	17.79±5.38	9.49	5.57	0.001^*^
	Clear aligners	8.00	2.70	16.30	8.30±3.83			
PD (mm)	Fixed appliance	1.30	0.44	1.68	1.25±0.34	0.37	3.10	0.001^*^
	Clear aligners	0.88	0.55	1.43	0.89±0.30			

The fixed appliance group showed significantly higher bacterial loads than the clear aligner group for both periodontal pathogens. *P. gingivalis* levels were markedly elevated in fixed appliances (median=150.5×10⁴ copies/mL) compared to clear aligners (median=98×10⁴ copies/mL). Similarly, *A. actinomycetemcomitans* was more prevalent in fixed appliances (median=5×10⁴ copies/mL) versus clear aligners (median=0×10⁴ copies/mL). These findings suggest that fixed appliances promote greater pathogenic colonization than aligners, potentially increasing the risk of periodontal disease. These results reinforce that clear aligners are a microbiologically favorable option, with significantly lower levels of key periodontopathogens (Table 4).

**Table 4:** Comparison of change in bacterial load (T1-T0, ×10⁴ copies/mL) between study groups using the Mann-Whitney U test ^*^P<0.05 indicates statistical significance using the Mann-Whitney U test. The bacterial load values of P. gingivalis and A. actinomycetemcomitans represent the change from baseline (T0) to three months (T1). Data presented as mean±SD, median, and range. SD: standard deviation

Bacteria	Groups	Median	Range	Mean±SD	Mean rank	Z statistic	P-value
Porphyromonas gingivalis	Fixed appliance	150.5	45-568	194.5±123.65	38.13	-3.39	0.012^*^
	Clear aligners	98	23-213	105.6±51.28	22.87		
*Aggregatibacter actinomycetemcomitans*	Fixed appliance	5	0-27	6.87±8.08	36.33	-2.92	0.003^*^
	Clear aligners	0	0-12	1.7±3.34	24.67		

The fixed appliance group demonstrated significantly higher microbial accumulation than the clear aligner group, particularly in the second premolars. *P. gingivalis* showed greater colonization with fixed appliances. *A. actinomycetemcomitans* was elevated in the fixed appliance group in the second premolar regions (median=9.53×10⁴ copies/mL in fixed appliances vs. 1.67×10⁴ copies/mL in clear aligners) but showed no significant difference in the lateral incisor region (p=0.121). These findings suggest that fixed appliances promote more pathogenic biofilms, especially in the posterior regions, potentially increasing periodontal risk. Overall, clear aligners maintained lower pathogen levels, reinforcing their microbiological advantage (Table 5).

**Table 5:** Comparison of change in bacterial load (T1-T0, ×10⁴ copies/mL) on specific teeth between study groups using the Mann-Whitney U test ^*^P<0.05 indicates statistical significance using the Mann-Whitney U test. The bacterial load values of P. gingivalis and A. actinomycetemcomitans represent the change from baseline (T0) to three months (T1). Data presented as median and range.

Bacteria	Group	Second premolars		Z statistic	P-value	Lateral incisors		Z statistic	P-value
		Median	Range			Median	Range		
P. gingivalis	Fixed appliance	234	122-568	-3.55	0.001^*^	112	45-178	-3.10	0.002^*^
	Clear aligners	134	78-213			67	23-134		
A. actinomycetemcomitans	Fixed appliance	9.53	0-27	-2.55	0.011^*^	4.20	0-16	-1.55	0.121
	Clear aligners	1.67	0-12			1.73	0-8		

## Discussion

This study aimed to quantify the change in the relative abundance of *P. gingivalis* and *A. actinomycetemcomitans* in subgingival plaque among orthodontic patients treated with fixed appliances versus clear aligners over a three-month period. Our results clearly demonstrated that fixed appliances were associated with significantly greater increases in these periodontal pathogens than clear aligners, underscoring the striking microbiological differences attributable to appliance type.

These findings are consistent with prior research demonstrating that fixed orthodontic appliances tend to foster greater plaque retention and shift toward pathogenic microbiota [5-10]. For instance, a study comparing fixed appliances to clear aligners within the same patients reported a ±10% increase in PI and increased colonization of gram-negative bacteria (including *P. gingivalis*) with fixed appliances, whereas aligner-treated arches maintained stable conditions or improved plaque levels [15]. Similarly, aligners have consistently been shown to promote superior periodontal health compared to fixed appliances, especially in terms of reduced plaque accumulation, gingival inflammation, and overall oral hygiene, across a range of observational and meta-analytic studies [10,11,16]. Clear aligners cover a significant portion of the dental crown, thereby inhibiting the accumulation of biofilms that can be removed, permitting patients to execute their oral hygiene practices under optimal circumstances [17].

Mechanistically, fixed appliances introduce numerous bracket-wire interfaces and difficult-to-reach surfaces that impede effective biofilm removal. The resulting microbial niches support anaerobic, gram-negative pathogens, such as *P. gingivalis *and *A. actinomycetemcomitans*. In contrast, clear aligners are removable, allowing patients to maintain routine hygiene with greater ease, which likely explains the relatively attenuated bacterial load increase observed in this group [18]. The existing body of literature has extensively examined the heightened susceptibility to white spot lesions and dental caries concomitant with orthodontic multibracket therapy, establishing elevated concentrations of *Streptococcus mutans*, *Streptococcus salivarius*, and *Lactobacilli* three months after the initiation of treatment [19,20]. One umbrella review further confirmed that aligners tend to result in better control of periodontal inflammation and probing depth, even if some of the numerical differences are small [16].

Our findings reinforce these trends at the microbial level: after three months, the median *P. gingivalis* load increased substantially more in the fixed appliance group, and *A. actinomycetemcomitans* followed a similar pattern. This suggests that fixed appliances may create an environment conducive to colonization by these key periodontopathogens, potentially increasing the risk of early periodontal deterioration. Furthermore, tooth-specific analyses revealed that the second premolars had particularly pronounced increases in bacterial load with fixed appliances compared with the lateral incisors. Anatomically, posterior teeth often present additional hygiene challenges, compounded by bracket complexity and saliva pooling, which may amplify bacterial retention and growth in those areas [21].

Importantly, these microbial findings have clinical relevance given the established role of these pathogens in initiating and sustaining periodontal inflammation and tissue destruction. Early periodontal dysbiosis, as evidenced here, can translate into heightened plaque accumulation, increased BOP, and depth gains if left unchecked. Indeed, our clinical data showed that fixed appliances were associated with worse periodontal changes in PI, BOP, and PD than clear aligners, which is consistent with the microbiological differences observed. Recent empirical investigations have demonstrated that the exacerbation of periodontal PD values throughout orthodontic interventions is predominantly attributable to inflammation of gingival tissues induced by bacterial biofilms, a condition that may culminate in gingival hyperplasia and formation of periodontal pseudopockets [7,22].

Throughout the course of fixed orthodontic treatment, it is imperative that dental hygienists are equipped with appropriate instruments to facilitate consistent and effective oral hygiene practices at home [23]. Tailored educational interventions, along with clinical and motivational strategies, should be employed to enhance the patient's understanding of the significance of regular tooth brushing in preserving optimal health for both dentition and gingival tissue, which is particularly vital for individuals undergoing fixed orthodontic therapy [24]. One prospective clinical trial found no difference in periodontal health outcomes between fixed and clear aligner patients when both groups received supportive periodontal therapy [25]. These findings highlight the critical role of adherence to hygiene and professional support during fixed orthodontic treatment.

Clinical implications

The present findings have important clinical relevance, particularly for orthodontic treatment planning in patients at risk of periodontal disease. Clear aligners appear to provide a microbiological and periodontal advantage by supporting better plaque control and reducing the colonization of periodontopathogens, such as *P. gingivalis* and *A. actinomycetemcomitans*. This suggests that clear aligners may be a more favorable option for adults or patients with a compromised periodontal status. However, for patients undergoing treatment with fixed appliances, the results emphasize the need for more rigorous monitoring, individualized hygiene instructions, and possibly adjunctive antimicrobial strategies to counteract the microbial shifts that occur around the brackets and wires. Greater attention should be directed to the posterior regions, where microbial accumulation is the most pronounced. Establishing optimal oral hygiene practices prior to appliance placement and maintaining strict compliance throughout treatment are crucial for minimizing the periodontal risk in orthodontic patients.

Limitations

This study has some limitations that should be acknowledged when interpreting the findings. First, the relatively short follow-up period of three months captures only the early phase of treatment and does not reflect the long-term microbial dynamics or periodontal changes that may emerge with prolonged appliance use. Second, the analysis was limited to two specific pathogens. Although these microorganisms are clinically significant, they do not encompass the full spectrum of microbial alterations associated with orthodontic appliances. The single-center design and modest sample size may also restrict the generalizability of our results to a broader population. In addition, while oral hygiene practices were standardized and monitored, actual patient compliance could not be completely controlled, and self-reported logs were inherently subject to bias. Finally, the non-randomized nature of the group allocation may have introduced potential confounding factors. Despite these limitations, this study provides valuable insights into the early microbial impact of orthodontic appliances and highlights the importance of incorporating periodontal considerations into appliance selection and treatment planning.

## Conclusions

Within the limitations of this study, it can be concluded that fixed orthodontic appliances were associated with a significantly greater increase in periodontal pathogens and deterioration of periodontal parameters over a three-month treatment period than clear aligners. The fixed appliance group demonstrated higher loads of *P. gingivalis *and *A. actinomycetemcomitans*, along with more pronounced increases in PI, BOP, and PD, whereas the clear aligner group maintained comparatively favorable periodontal and microbial profiles. These findings suggest that clear aligners may be the preferred treatment modality for patients in whom periodontal health preservation is a priority.
